# An energy-aware routing method using firefly algorithm for flying ad hoc networks

**DOI:** 10.1038/s41598-023-27567-7

**Published:** 2023-01-24

**Authors:** Jan Lansky, Amir Masoud Rahmani, Mazhar Hussain Malik, Efat Yousefpoor, Mohammad Sadegh Yousefpoor, Muhammad Umair Khan, Mehdi Hosseinzadeh

**Affiliations:** 1 Department of Computer Science and Mathematics, Faculty of Economic Studies, University of Finance and Administration, Prague, Czech Republic; 2 Future Technology Research Center, National Yunlin University of Science and Technology, 123 University Road, Section 3, 64002, Douliou, Yunlin, Taiwan; 3 School of Computing and Creative Technologies, College of Arts, Technology and Environment (CATE), University of the West of England, Frenchay Campus, Coldharbour Lane, Bristol BS16 1QY, United Kingdom; 4 Department of Computer Engineering, Dezful Branch, Islamic Azad University, Dezful, Iran; 5 School of Computing, Gachon University, 1342, Seongnam-daero, Sujeong-gu, Seongnam-si 13120, Republic of Korea; 6 Institute of Research and Development, Duy Tan University, Da Nang, Vietnam; 7 Computer Science, University of Human Development, Sulaymaniyah, Iraq

**Keywords:** Engineering, Mathematics and computing, Physics

## Abstract

Flying ad-hoc networks (FANETs) include a large number of drones, which communicate with each other based on an ad hoc model. These networks provide new opportunities for various applications such as military, industrial, and civilian applications. However, FANETs have faced with many challenges like high-speed nodes, low density, and rapid changes in the topology. As a result, routing is a challenging issue in these networks. In this paper, we propose an energy-aware routing scheme in FANETs. This scheme is inspired by the optimized link state routing (OLSR). In the proposed routing scheme, we estimate the connection quality between two flying nodes using a new technique, which utilizes two parameters, including ratio of sent/received of hello packets and connection time. Also, our proposed method selects multipoint relays (MPRs) using the firefly algorithm. It chooses a node with high residual energy, high connection quality, more neighborhood degree, and higher willingness as MPR. Finally, our proposed scheme creates routes between different nodes based on energy and connection quality. Our proposed routing scheme is simulated using the network simulator version 3 (NS3). We compare its simulation results with the greedy optimized link state routing (G-OLSR) and the optimized link state routing (OLSR). These results show that our method outperforms G-OLSR and OLSR in terms of delay, packet delivery rate, throughput, and energy consumption. However, our proposed routing scheme increases slightly routing overhead compared to G-OLSR.

## Introduction

Unmanned ariel vehicles (UAVs) or drones are aircraft driven by embedded programs or remote control. At the first time in 1930, the US military used UAVs for airstrikes, reconnaissance, and monitoring boundaries. Next, in 1990, UAVs have widely been used in many military and civilian applications^1,2^. Usually, UAVs are divided into two types: fixed-wings and rotary-wings. Rotary-wing UAVs provide vertical landing and take-off and can stay fixed in the air. This feature helps them to be used in sensing tasks. However, they have less energy, limited speed, and low capacity^3,4^. Fixed-wing UAVs can move on the air and cannot be fixed in the air. They fly faster and have suitable capacity. Also, they are energy-efficient and can carry heavier payload. They can be used for monitoring the airborne. They are more expensive than rotary-wing UAVs^3,5^.

Moreover, drones are created in different sizes from small to large. According to the size of drones, they can be used in specific applications^6,7^. For example, drones with large sizes are used in military missions. In contrast, drones with small sizes are usually used in civilian applications^8,9^. Drones with small sizes include cheap bodies, small batteries, small radio equipment, and microprocessors^10^. Also, they need low costs for repair and maintenance. However, drones with large sizes have more abilities^11,12^. Also, they have a high weight and payload. When several drones work together in an ad hoc manner, they build a new ad hoc network called flying ad-hoc network (FANET)^13,14^. In FANETs, a group of UAVs communicate with each other without any access point. However, at least one of UAVs should communicate with ground station (GS) or satellite^15,16^. In fact, this network is known as a subset of mobile ad hoc network (MANET) and vehicular ad Hoc network (VANET)^17,18^. Today, FANETs have provided new opportunities to improve many applications, for example, monitoring the environment, improving urban traffic through smart monitoring, air imaging, search and rescue operations, agricultural operations, and monitoring nuclear powerhouse. However, these networks have many challenges like highly dynamic topology, high-speed of nodes, low density. As a result, routing is a challenging issue in these networks because UAVs have communication links with a short lifetime^19,20^. Furthermore, drones with small sizes have limited energy due to their structural features. Therefore, this issue must be considered when designing a routing scheme for this network^21,22^. The most common routing protocols are static routing^23,24^, proactive routing^23,24^, reactive routing^23,24^, hybrid routing^25,26^, geographical routing^23,24^, and multi-path routing^25,27^. The performance of a routing protocol depends on selecting the optimal route, which can be calculated based on various criteria, for example, speed, energy, link lifetime, network traffic, delay, link quality, and movement direction^25,28^.

In this paper, we propose an energy-aware routing scheme for FANETs. This scheme is inspired by the optimized link state routing (OLSR). In the first step, we present a new technique for calculating connection quality. It is calculated based on ratio of sent/received of hello packets and connection time. We use this parameter for calculating optimal routes. This helps our scheme to select more stable routes. Furthermore, we propose a new approach based on firefly algorithm (FA)^29^ to select multipoint relays (MPRs). This approach is called MPR-FA. According to MPR-FA, a symmetric and single-hop neighboring node with more energy, higher connection quality, more neighborhood degree, and higher willingness achieves more chance to be selected as MPR. The major contributions of our proposed scheme are summarized as follows:

- Improving the *Hello message* format and the *topology control (TC) message* format.
- Proposing a new scheme for calculating the connection quality.
- Selecting MPR nodes using the firefly algorithm.
- Calculating the routing table based on energy and connection quality.

The rest of the paper is organized as follows: In “Related works” section, we examine various routing methods presented in FANETs. In “Basic concepts” section, we define the concepts related to the firefly algorithm, which is used in the proposed method. In “Network model” section, we present the network model used in our method. In “Proposed method” section, we describe the proposed routing method in detail. In “Simulation and evaluation of results” section, we simulate the proposed method and compare its results with G-OLSR and OLSR. In “Conclusion” section, we conclude the paper.

## Related works

Lee et al.^30^ suggested an energy-aware and predictive fuzzy logic-based routing scheme in FANETs. This approach includes two processes: route discovery and route maintenance. In the route discovery process, each UAV calculates its score based on movement direction, remaining energy, link quality, and node stability. If the node achieves enough score, it can participate in rebroadcasting route request messages (RREQs) and otherwise, it is not allowed to do this. The purpose of this work is to balance energy consumption, reduce congestion in the network, and control the broadcast storm problem. When RREQ messages arrive at the destination, the destination node selects the best path to the source node using the fuzzy system. The best path is a route with a minimum number of hops, low delay, and high fitness. In the route maintenance process, two issues are addressed: preventing path failure and rebuilding failed paths. This method creates stable routes between network nodes. Also, this scheme is simulated in a 3D space. However, the route maintenance process is slightly ambiguous and can be improved.

Mahmoud and Cho^31^ offered a location estimation-based congestion-aware routing method (LECAR) for FANETs. It is a delay-tolerant network (DTN)-based routing scheme and tries to solve two problems in DTN-based routing schemes: high energy consumption and high network congestion. In this method, each node estimates its location and locations of other nodes using the path-planning mechanism and location map. In the routing process, the source node sends its data packet to the node, which is nearer to the destination and has enough buffer capacity. This method successfully balances energy consumption in the network and prevents network congestion. However, it has high communication overhead and delay. This method is simulated in a two-dimensional space that is not compatible with FANETs.

Clausen et al.^32^ presented the optimized link state routing protocol (OLSR) for mobile ad hoc networks (MANETs). It is one of the well-known proactive routing schemes. OLSR depends on multipoint relays (MPRs), which are tasked to produce and forward topology messages in the network. However, OLSR has several disadvantages, including high communication overhead and delay. Therefore, OLSR is not suitable for delay-intolerant applications. Today, many researchers focus on OLSR to modify this protocol for FANETs.

For example, Ali et al.^33^ proposed the greedy optimized link state routing (G-OLSR) scheme for FANETs. G-OLSR merges two methods, including the greedy perimeter stateless routing (GPSR) and OLSR. G-OLSR minimizes communication overhead and delay in the network. As a result, it is suitable for delay-intolerant applications. This method utilizes a self-adaptation approach when varying network topology. This enhances the network performance. A greedy approach is applied for choosing the next-hop node. In this approach, the next-hop node is selected based on maximum distance from the source toward the destination. When the greedy approach cannot find the next-hop node, G-OLSR switches to the route recovery mode. This case occurs when there is no node whose distance toward the destination node is longer than the previous-hop node. In the recovery mode, G-OLSR considers the angle between a neighboring node and the destination node to select the next-hop node. This method is simulated in a 3D space, which is suitable for FANET. However, G-OLSR ignores the energy and link quality in the routing process. In G-OLSR, unstable paths may be created.

Rahmani et al.^34^ presented a fuzzy-based routing protocol called OLSR+ for flying ad hoc networks. It is an improved version of the optimized link state routing protocol (OLSR). In this method, the authors proposed a new technique to calculate the lifetime of the link between two UAVs. The link lifetime is obtained based on the received signal strength indication (RSSI), Euclidean distance, relative velocity, and motion direction. Then, MPRs are selected using a fuzzy system. This fuzzy system consists of three inputs, including the residual energy of a neighboring UAV, link lifetime, and the degree of a neighboring UAV. The fuzzy output indicates the chance of a neighboring node to be selected as MPR. Finally, the routing paths are determined between the nodes based on three parameters, including the number of hops, route energy, and route lifetime. The simulation results indicate that OLSR+ has a successful performance in terms of delay and energy consumption. However, OLSR+ has a lot of communication overhead.

Ma et al.^35^ offered the velocity-aware and stability-estimation-based multi-path routing protocol (VaSe-MRP) in FANETs. This routing scheme includes two steps: route discovery and route maintenance. In the first step, the authors present a forwarding mechanism, which is based on the velocity of nodes and categorizes the nodes into two classes: reliable class and non-reliable class. Next-hop nodes are selected from reliable class. Also, VaSe-MRP calculates the link stability time and selects several routes between transmitter and receiver. Therefore, VaSe-MRP increases packet delivery rate. However, it is implemented in a 2D space, which is not suitable for FANET. VaSe-MRP considers a very low speed for nodes when simulating this scheme. Therefore, it must be evaluated for high-speed nodes.

Chen et al.^36^ suggested the traffic-aware Q-network enhanced geographic routing scheme (TQNGPSR). TQNGPSR presents a new mechanism based on congestion information. This mechanism balances network traffic. Furthermore, this scheme utilizes the Q-network reinforcement algorithm to analyze link quality according to the congestion information. In fact, the Q-value represents the link quality. Finally, TQNGPSR chooses routes based on Q-value. These routes include low delay, low packet loss rate, and low traffic. However, TQNGPSR is implemented in a 2D space, which is a false option for FANETs. This scheme does not consider the energy parameter and link lifetime. However, they are very important when designing a routing protocol in FANETs. Also, it includes a high communication overhead.

Oubbati et al.^37^ proposed the energy-efficient connectivity-aware data delivery (ECaD) for FANETs. This scheme prevents route failure and reconstructs failed routes. In ECaD, the energy parameter, link lifetime, and delay are considered in the routing process. If UAVs have enough energy, they can rebroadcast RREQ messages and otherwise, they do not participate in this process. This work balances energy consumption in the network. It is simulated in a 3D space, which is a suitable option for FANET. However, ECaD ignores the movement direction and the link quality when selecting routes.

Liu et al.^38^ proposed the Q-learning-based multi-objective routing protocol (QMR) for FANETs. QMR utilizes a reinforcement algorithm (Q-learning) for finding paths. Firstly, QMR sets the learning rate and the discount factor in Q-learning algorithm according to network conditions. This scheme decreases delay and energy consumption. In QMR, each node estimates the future status of its neighbors in the network. This advantage helps nodes to choose the best next-hop nodes. QMR considers various parameters like, speed, movement direction, and delay in the routing process. This scheme builds stable route in the network. Furthermore, QMR is simulated in a 2D space, which is a false option for FANET. In addition, QMR predicts route failure and rebuilds failed routes. However, QMR has a major disadvantage. It cannot control swarm connectivity.

## Basic concepts

In this section, we explain the firefly algorithm (FA) briefly because this concept is used in our approach for selecting MPR nodes.

### Firefly algorithm

In late 2007, Xin-She Yang introduced the firefly algorithm (FA). FA is inspired by optical patterns and the behavior of fireflies^29^. FA is based on several rules:

- Fireflies are single-sex. This means that fireflies are attracted to each other without considering their sex.
- Attractiveness of fireflies is defined based on their brightness. This means that the less bright fireflies are attracted to brighter fireflies. When the distance between fireflies increases, their attractiveness decreases. If there is not a brighter firefly, the movement of fireflies is random.
- An objective function is defined for calculating the brightness of fireflies.

FA considers two parameters: the variation of light intensity and attractiveness. Attractiveness is obtained based on brightness. Furthermore, brightness is defined based an objective function.

Brightness of a firefly (i.e. *I*) at a specific position *x* is $I(x) \propto f(x)$. However, attractiveness (i.e. $\beta$) ia relative and depends on the distance $r_{ij}$ between $firefly_{i}$ and $firefly_{j}$. Moreover, if firefly is far from the light source, the light intensity reduces. Furthermore, the media absorbs light. Moreover, attractiveness depends on the absorption degree. Usually, the light intensity *I*(*r*) varies according to the inverse-square law expressed in Eq. (1):

1 $$\begin{aligned} I(r)=\frac{I_{s}}{r^{2}}, \end{aligned}$$

where, $I_{s}$ indicates the intensity at the source.

Assume a specific medium, which has a constant light absorption coefficient $\gamma$, Eq. (2) computes *I* according to the distance *r*:

2 $$\begin{aligned} I(r)=I_{0}e^{-\gamma r}, \end{aligned}$$

where, $I_{0}$ indicates the initial light intensity at $r=0$. Eqs. (1) and (2) are estimated as Eq. (3) to prevent the singularity at $r=0$ in the phrase $\frac{I_{s}}{r^{2}}$:

3 $$\begin{aligned} I(r)=I_{0}e^{-\gamma r^{2}}. \end{aligned}$$

Also, Eq. (4) computes $\beta$ of a firefly:

4 $$\begin{aligned} \beta =\beta _{0}e^{-\gamma r^{2}}, \end{aligned}$$

where, $\beta _{0}$ is the attractiveness at $r=0$. Equation (4) is estimated as Eq. (5) because $\frac{1}{1+r^{2}}$ is computed faster than an exponential function:

5 $$\begin{aligned} \beta =\frac{\beta _{0}}{1+\gamma r^{2}}. \end{aligned}$$

Equation (6) expresses the distance between $firefly_{i}$ and $firefly_{j}$ at $z_{i}$ and $z_{j}$, respectively:

6 $$\begin{aligned} r_{ij}=\Vert z_{i}-z_{j}\Vert =\sqrt{\sum _{k=1}^{d}(z_{i,k}-z_{j,k})^{2}}, \end{aligned}$$

where, $z_{i,k}$, is the *k*th component of the spatial coordinate $z_{i}$ of *i*th firefly.

According to Eq. (7), the $firefly_{i}$ is attracted to more attractive $firefly_{j}$:

7 $$\begin{aligned} z_{i}^{t+1} =z_{i}^{t}+\beta _{0}e^{-\gamma r_{ij}^{2}}(z_{j}^{t}-z_{i}^{t})+\alpha \varepsilon _{i}^{t}, \end{aligned}$$

where, attraction is expressed in the second phrase. Also, randomization is created using the third phrase. It is achieved through the randomization parameter $\alpha$. And, *i* indicates a vector of random numbers. Note that, $\epsilon _{i}$ can be replaced with $rand\frac{-1}{2}$ so that *rand* generates a random number in [0, 1]. Generally, it is assumed that $\beta _{0}=1$ and $\alpha \in [0,1]$. For more details, please refer to Ref.^29^. We use FA to select MPR nodes in the network because this algorithm can satisfactorily explore the optimal global response. Also, FA has a high convergence speed and acceptable accuracy. Furthermore, it can make a balance between global search and local search and is implemented easily.

## Network model

**Figure 1:**
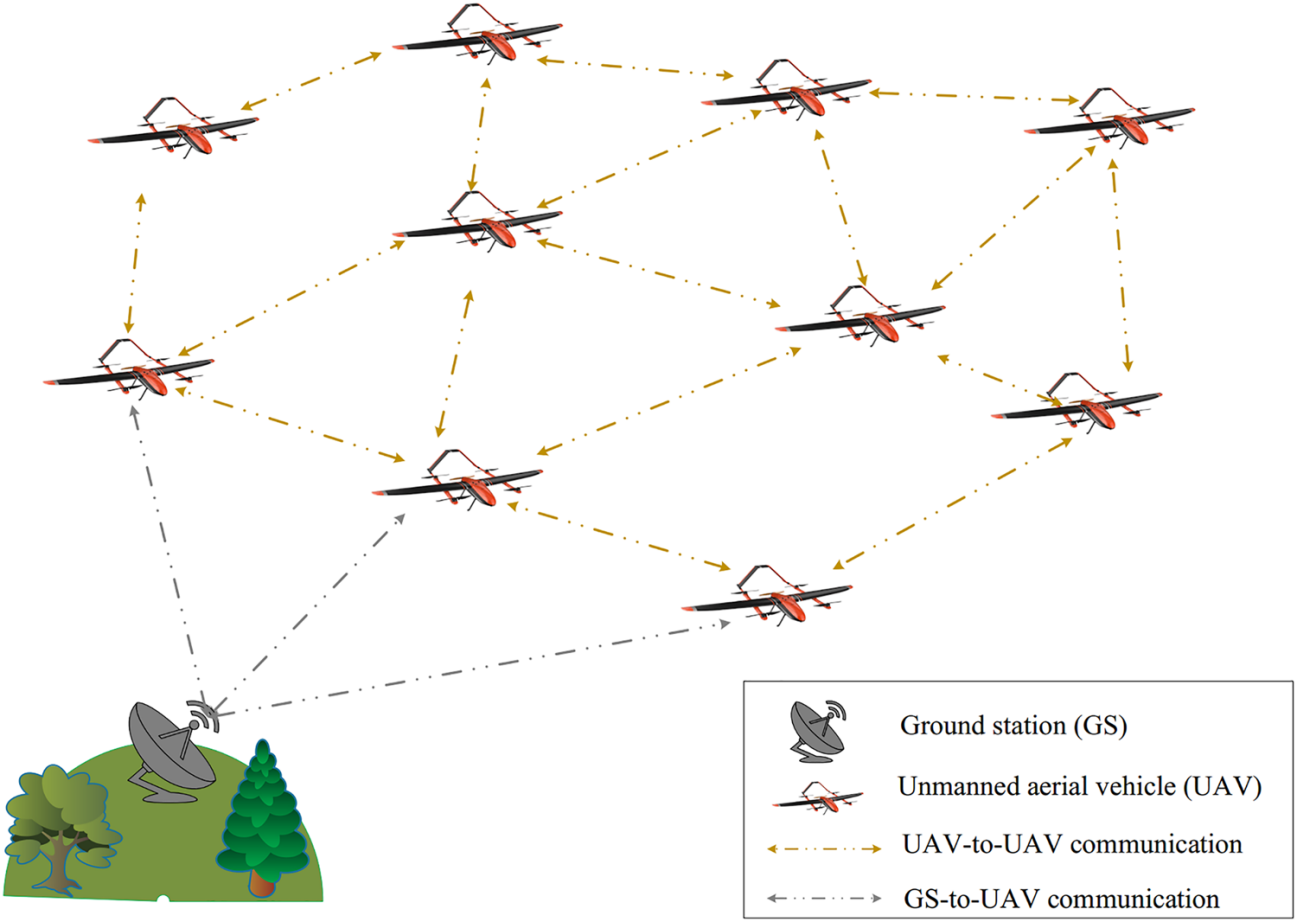
Network model in the proposed scheme.

In the proposed routing method, we assume that the network includes *N* nodes such as $UAV_{i},\,\,\,\,i=1,2,\ldots ,N$. Each node has a unique identifier ($ID_{UAV_{i}}$). These nodes are randomly distributed in FANET. FANET is a three-dimensional environment and the position of nodes is represented based on length, width, and height. Each node is connected to the global positioning system (GPS) to know its position $\left( x_{i}^{t},y_{i}^{t},z_{i}^{t} \right)$ and its speed $\left( v_{x,i}^{t},v_{y,i}^{t},v_{z,i}^{t} \right)$ at moment *t*. Also, nodes move at a speed ${\textbf{V}_{UAV}}$ in the network. Therefore, the network topology changes continuously and communication links fail. In addition, it is assumed that the network supports UAV to UAV communications (U2U) and UAV to GS communications (U2G). In U2G communication, a small number of UAVs communicate directly with GS, other UAVs act as a relay node and transmit data packets in a multi-hop manner. We use the IEEE 802.11a protocol as a wireless interface in the MAC layer of each UAV because it provides appropriate bandwidth. Also, it supports high-dynamic topologies and provides a wide range of wireless communication. The network model is shown in Fig. 1.

## Proposed method

In this paper, we propose an energy-aware routing scheme for FANETs. This method is inspired by OLSR. The proposed scheme includes five steps:

- Improving the *Hello message* format and the neighborhood table.
- Calculating the connection quality.
- Introducing the MPR-FA approach to select MPRs.
- Modifying the *TC message* format.
- Computing different routes.

In the following, these steps are described in detail. Moreover, we present the most important symbols used in our scheme in Table 1.

**Table 1:** Symbols used in the paper.

Symbols	Definition
*N*	The total number of UAVs
$ID_{UAV_{i}}$	ID of $UAV_{i}$
$\left( x_{i}^{t},y_{i}^{t},z_{i}^{t} \right)$	Spatial coordinates of $UAV_{i}$ at moment *t*
$\left( v_{x,i}^{t},v_{y,i}^{t},v_{z,i}^{t} \right)$	Velocity of $UAV_{i}$ at moment *t*
$CQ_{ij}$	The quality of the link between $UAV_{i}$ and $UAV_{j}$
$CQ_{ij}^{initial}$	The quality of the initial connection between $UAV_{i}$ and $UAV_{j}$
$CT_{ij}\left( t \right)$	Connection time of link between $UAV_{i}$ and $UAV_{j}$
$\Delta {{\theta }_{ij}}$	Movement direction of $UAV_{j}$ with regard to $UAV_{i}$
*R*	Communication radius of UAVs
$\textbf{V}_{ij}$	Relative velocity of $UAV_{j}$ with respect to $UAV_{i}$
$UAV_{MPR}$	MPR nodes in the network
$SET_{MPR}(UAV_{i})$	A set of MPR nodes
$List_{2Hop{\text{-}}Neighbor}$	A list of two-hop neighbors of ${UAV_{i}}$
$W_{i}$	Willingness of a node to carry and send traffic to other nodes
$D_{UAV_{i}}$	The degree of ${UAV_{i}}$ or the number of its symmetric neighbors
$List_{One{\text{-}}hop{\text{-}}neighbor}$	A list of all symmetric single-hop neighbors of ${UAV_{i}}$
$E_{UAV}$	The remaining energy of a UAV
$F_{j}$	The fitness value of $UAV_{j}$
$E_{Path}$	Path energy
$CQ_{Path}$	Path quality

### Hello message format and neighborhood table

**Figure 2:**
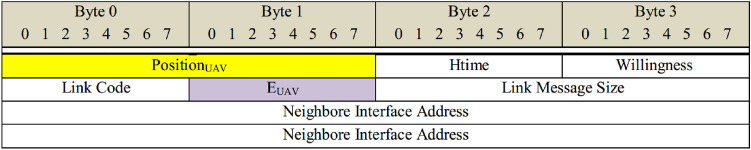
*Hello message* format.

A *Hello message* is sent to its neighboring UAVs by each $UAV_{i}$ ($i=1,\ldots ,N$). After receiving this message, $UAV_{i}$ knows all its own single-hop and two-hop neighboring UAVs in the network. Figure 2 displays the modified *Hello message* format. As shown in this figure, all fields of the modified message are similar to the original *Hello message*^32^ except two fields, $Position_{UAV}$ (Yellow color) and $E_{UAV}$ (Purple color). The $Position_{UAV}$ field indicates spatial coordinates of $UAV_{i}$, which is obtained using GPS. The $E_{UAV}$ field represents the remaining energy of $UAV_{i}$.

According to *Hello message* received from neighboring UAVs, a neighborhood table is built in $UAV_{i}$. For two-hop neighbors, the format of this table is similar to OLSR. But, for single-hop neighbors, this table maintains their main address, their status (symmetric or non-symmetric), their willingness value, their position, their velocity, their remaining energy, and their connection quality. In the following, we describe the most important parameters for single-hop neighbors:

- *Willingness* $UAV_{i}$ obtains this parameter from the *Hello message*. It represents the willingness of a node to carry and send traffic to other nodes. It is a integer number in [0, 7].
- *Position* $UAV_{i}$ extracts the spatial coordinates of its neighboring UAV from the $Position_{UAV}$ field in the *Hello message*.
- *Velocity* $UAV_{i}$ calculates the velocity of a single-hop neighboring node such as $UAV_{j}$ using the $Position_{UAV}$ field in two sequential *Hello messages*. This is expressed in Eq. (8):
  8 $$\begin{aligned} V_{j}=\frac{\sqrt{{{\left( x_{j}^{p}-{x_{j}^{p-1}}\right) }^{2}}+{{\left( y_{j}^{p}-{y_{j}^{p-1}} \right) }^{2}}+{{\left( z_{j}^{p}-{z_{j}^{p-1}}\right) }^{2}}}}{{t_{p}}-{t_{p-1}}}, \end{aligned}$$
  where, $\left( x_{j}^{p},y_{j}^{p},z_{j}^{p}\right)$ represents the spatial coordinates of $UAV_{j}$ at the moment $t_{p}$ and $\left( {x_{j}^{p-1}},{y_{j}^{p-1}},{z_{j}^{p-1}}\right)$ is the spatial coordinates of $UAV_{j}$ at the moment $t_{p-1}$.
- *Remaining energy* $UAV_{i}$ extracts the remaining energy of its neighbors from the $E_{UAV}$ field in the *Hello message*.
- *Connection qualit *(${{\text {CQ}}_\text {ij}}$) $UAV_{i}$ calculates this parameter according to Eq. (10) described in “Calculatingtheconnectionquality” section.

### Calculating the connection quality

In a dynamic environment such as FANET, the quality of the connection between two nodes cannot be easily evaluated because the nodes are highly moving and their connection quality changes over time. Therefore, we estimate the quality of the connection between $UAV_{i}$ and $UAV_{j}$ (which is called $CQ_{ij}$) using an approximate model. According to this model, $UAV_{i}$ measures the quality of the initial connection between itself and $UAV_{j}$ (which is called $CQ_{ij}^{initial}$) based on the ratio of Hello packets received by $UAV_{i}$ to the total messages transmitted from $UAV_{j}$ to $UAV_{i}$ at a time interval ($\tau$). Note that each node uses a counter for counting the number of Hello messages received from its neighbors within its communication range. Also, we know that *Hello messages* are sent in a certain time period (for example, one second), so we can obtain the number of sent messages in a time interval. As a result, each node calculates the Hello packet reception rate to evaluate the connection quality. $CQ_{ij}^{initial}$ is calculated according to Eq. (9):

9 $$\begin{aligned} CQ_{ij}^{initial}=\frac{\sum \nolimits _{t\in \tau }{{{R}_{hello}}\left( i,j \right) }}{\sum \nolimits _{t\in \tau }{{{S}_{hello}}\left( i,j \right) }}, \end{aligned}$$

where, ${{R}_{hello}}\left( i,j \right)$ and ${{S}_{hello}}\left( i,j \right)$ indicate the number of Hello messages received by $UAV_{i}$ and the number of Hello messages sent from $UAV_{j}$ to $UAV_{i}$ in the time period $\tau$, respectively.

Note that the connection quality is not a fixed parameter and changes over time. To evaluate the quality of the connection between two nodes at any moment, the connection quality is updated based on the connection time. Therefore, $C{{Q}_{ij}}\left( t \right)$ is calculated at the moment *t*, according to Eq. (10):

10 $$\begin{aligned} C{{Q}_{ij}}\left( t \right) =CQ_{ij}^{initial}{{e}^{-\left( 1-C{{T}_{ij}}\left( t \right) \right) }}, \end{aligned}$$

where, $CT_{ij}\left( t \right)$ indicates the connection time of link between $UAV_{i}$ and $UAV_{j}$. $CT_{ij}\left( t\right)$ is calculated using Eq. (11). According to Eq. (10), if $CT_{ij}\left( t \right)$ is high (i.e. it is close to *one*), the connection quality is close to the initial connection quality. Also, when the connection time is low (i.e. it is close to *zero*), the connection quality will be lower than the initial connection quality.

In Eq. (11), the connection time between between $UAV_{i}$ and $UAV_{j}$ is calculated based on two parameters, including the Euclidean distance and relative velocity of $UAV_{j}$ with regard to $UAV_{i}$. In this equation, the movement direction is also considered.

11 $$\begin{aligned} C{{T}_{ij}}\left( t \right) =\frac{\left( \frac{R-{{\lambda }_{1}}\sqrt{{{\left( x_{i}^{t}-x_{j}^{t} \right) }^{2}}+{{\left( y_{i}^{t}-y_{j}^{t} \right) }^{2}}+{{\left( z_{i}^{t}-z_{j}^{t} \right) }^{2}}}}{\left| V_{i}^{t}-{{\lambda }_{2}}V_{j}^{t} \right| } \right) }{{{T}_{\max }}}, \end{aligned}$$

where, ${{T}_{\max }}$ indicates the maximum connection time. This parameter is a fixed value and is determined based on the simulation time. *R* is the communication radius of UAVs. Also, $\left( x_{i}^{t},y_{i}^{t},z_{i}^{t} \right)$ and $V_{i}^{t}$ indicate spatial coordinates and speed of $UAV_{i}$ at time *t*, respectively. $\left( x_{j}^{t},y_{j}^{t},z_{j}^{t} \right)$ and $V_{j}^{t}$ are spatial coordinates and speed of $UAV_{j}$ at the moment *t*, respectively. In addition, $\Delta {{\theta }_{ij}}$ is the movement direction of $UAV_{j}$ with regard to $UAV_{i}$. It is obtained according to Eq. (12):

12 $$\begin{aligned} \Delta {{\theta }_{ij}}={{\cos }^{-1}}\left( \frac{v_{x,i}^{t}v_{x,j}^{t}+v_{y,i}^{t}v_{y,i}^{t}+v_{z,i}^{t}v_{z,i}^{t}}{|V_{i}^{t}||V_{j}^{t}|} \right) ,\,\,\,0\le \Delta {{\theta }_{ij}}\le \pi , \end{aligned}$$

where, $\left( v_{x,i}^{t},v_{y,i}^{t},v_{z,i}^{t} \right)$ and $\left( v_{x,j}^{t},v_{y,j}^{t},v_{z,j}^{t} \right)$ indicate the velocity vectors of $UAV_{i}$ and $UAV_{j}$ at the moment *t*, respectively. Furthermore, $\left| V_{i}^{t}\right| =\sqrt{{{\left( v_{x,i}^{t} \right) }^{2}}+{{\left( v_{y,i}^{t} \right) }^{2}}+{{\left( v_{z,i}^{t} \right) }^{2}}}$ and $\left| V_{j}^{t}\right| =\sqrt{{{\left( v_{x,j}^{t} \right) }^{2}}+{{\left( v_{y,j}^{t} \right) }^{2}}+{{\left( v_{z,j}^{t} \right) }^{2}}}$.

**Figure 3:**
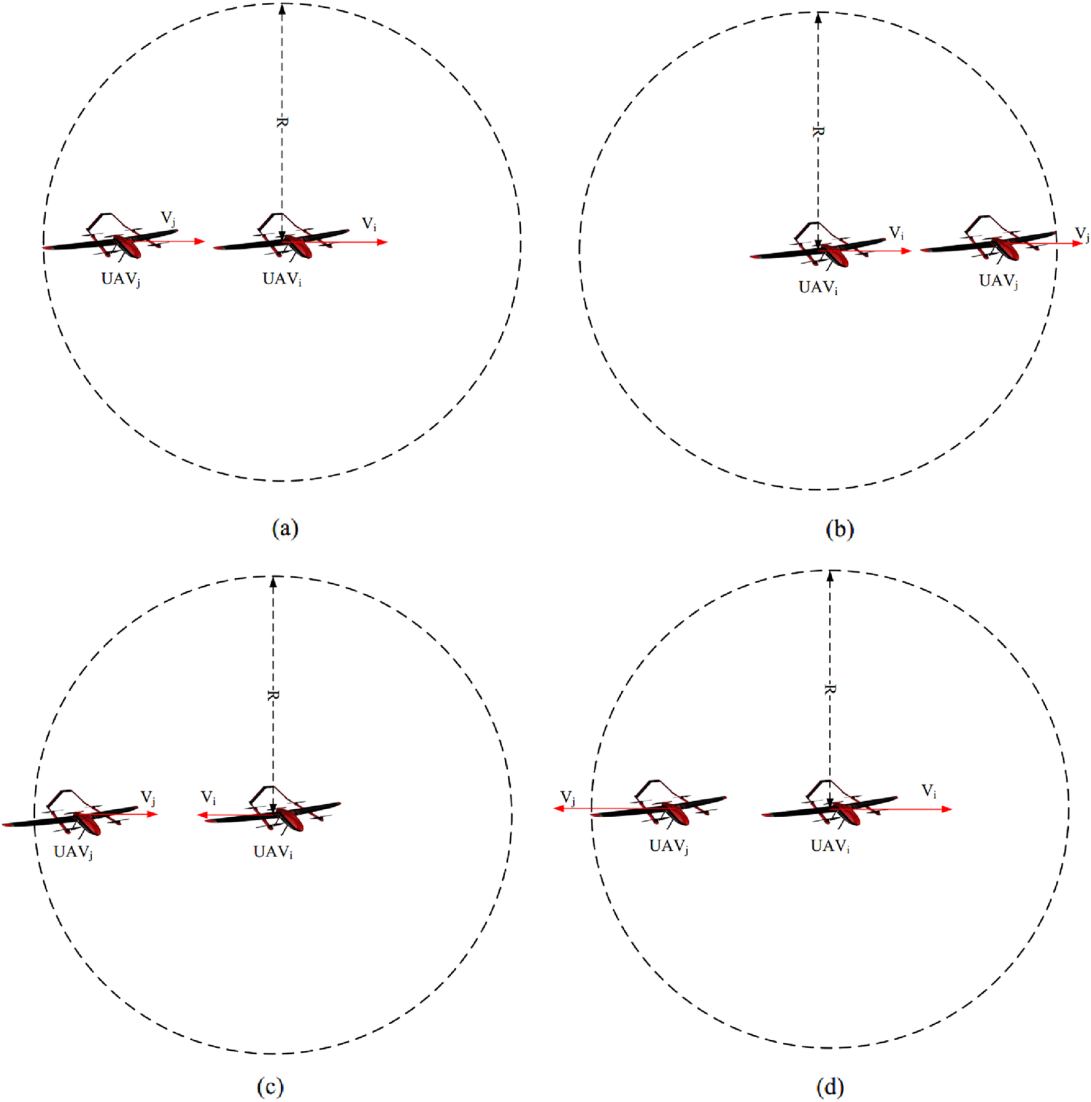
Various modes of $UAV_{i}$ and $UAV_{j}$.

Finally, ${{\lambda }_{1}}$ and ${{\lambda }_{2}}$ are configuration parameters in Eq. (11). These parameters are determined based on the four modes:

- *Mode 1* When $UAV_{i}$ and $UAV_{j}$ move in the same direction (i.e. $0\le \Delta {{\theta }_{ij}}\le \frac{\pi }{2}$), and $UAV_{i}$ is ahead of $UAV_{j}$ (see Fig. 3a). Then, ${{\lambda }_{1}}=1$ and ${{\lambda }_{2}}=1$.
- *Mode 2* When $UAV_{i}$ and $UAV_{j}$ move in the same direction (i.e. $0\le \Delta {{\theta }_{ij}}\le \frac{\pi }{2}$), and $UAV_{i}$ is behind $UAV_{j}$ (see Fig. 3b). Then, ${{\lambda }_{1}}=-1$ and ${{\lambda }_{2}}=1$.
- *Mode 3* When $UAV_{i}$ and $UAV_{j}$ move in the opposite direction (i.e. $\frac{\pi }{2}\le \Delta {{\theta }_{ij}}\le \pi$), and $UAV_{i}$ is approaching $UAV_{j}$ (see Fig. 3c). Then, ${{\lambda }_{1}}=-1$ and ${{\lambda }_{2}}=-1$.
- *Mode 4* When $UAV_{i}$ and $UAV_{j}$ move in the opposite direction (i.e. $\frac{\pi }{2}\le \Delta {{\theta }_{ij}}\le \pi$), and $UAV_{i}$ is going away from $UAV_{j}$ (see Fig. 3d). Then, ${{\lambda }_{1}}=1$ and ${{\lambda }_{2}}=- 1$.

### Selecting MPR nodes

**Figure 4:**
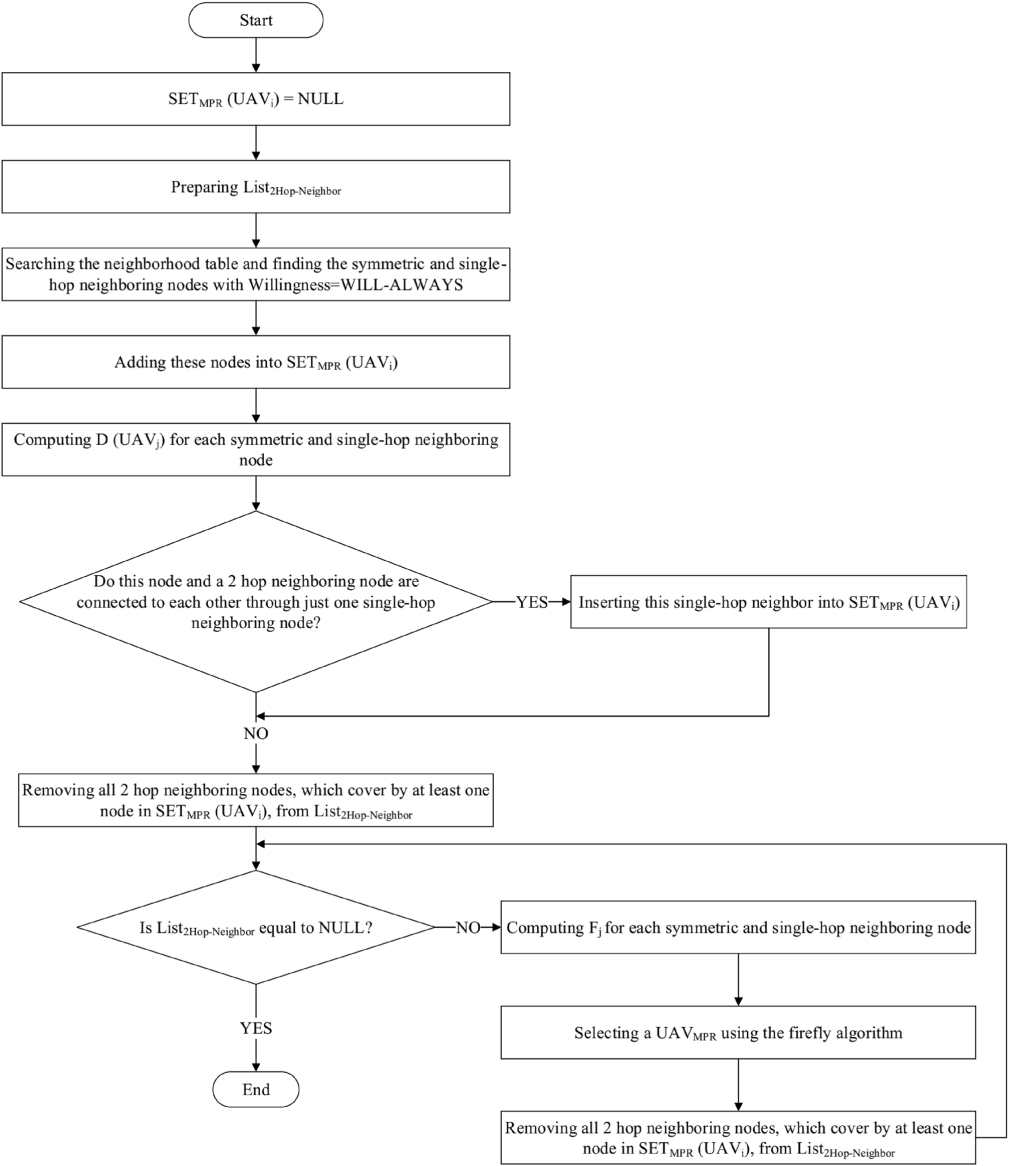
Flowchart of MPR node selection.

In this step, MPR nodes ($UAV_{MPR}$) are selected using the firefly algorithm. Each $UAV_{i}$ ($i=1,\ldots ,N$) must select a set of its neighbors as “multipoint relays”(MPRs). This set is called $SET_{MPR}\left( UAV_{i}\right)$. When calculating various routes, MPRs are responsible for creating the route from a source node to any destination in the network. This means that only these nodes can forward control message (TC message) into the network. This reduces the number of transmission when flooding control traffic. In this section, we present the MPR-FA approach based on firefly algorithm to select $UAV_{MPR}$ (see Fig. 4). In this process, we assume that each firefly expresses each symmetric single-hop neighbor of $UAV_{i}$ such as $UAV_{j}$. Firstly, a random value is assigned to each firefly. As a result, $UAV_{i}$ considers each of its symmetric single-hop neighbors as a firefly, which can be selected as $UAV_{MPR}$. *Rand* function determines the initial attractiveness of a firefly. The end condition for this algorithm is equal to 100 iterations. Finally, a firefly having the most fitness value is selected as a MPR node. In the proposed algorithm, each $UAV_{i}$ follows the following steps:

1. Consider each of its symmetric single-hop neighbors as a firefly.

2. Initialize the firefly population randomly.

3. Set $$SET_{MPR}\left( UAV_{i}\right) =\varnothing$$.

4. Prepare the two-hop neighbor list of $UAV_{i}$ ($List_{2Hop-Neighbor}$).

5. Insert the identifier of all symmetric single-hop neighbors of $UAV_{i}$, which their willingness is equal to “*WILL-ALWAYS*”, into $SET_{MPR}\left( UAV_{i}\right)$.

6. Calculate the degree of each single-hop neighbor (i.e. the number of its symmetric neighbors), like $UAV_{j}$ according to the information in its neighborhood table. This metric is represented as $D\left( UAV_{j}\right)$.

7. Insert the identifier of a single-hop neighbor into $SET_{MPR}\left( UAV_{i}\right)$, when $UAV_{i}$ can communicate with its two-hop neighbor only by this single-hop neighbor.

8. Remove identifiers of all two-hop neighbors from $List_{2hop{\text{-}}neighbor}$ when they are covered by at least one MPR.

9. Repeat the following steps until $List_{2hop{\text{-}}neighbor}=\varnothing$:

- Calculate the fitness function presented in Eq. (18) for each firefly. This function is calculated based on four parameters. We explain these parameters in the following:
  - ${\textit{E}}_{\textit{j}}^{\textit{Residual}}$ This metric indicate the remaining energy of each firefly at any moment to determine how much the UAV has the ability to manage the required network tasks and can continue its activity in the network. This metric helps $UAV_{i}$ to choose high-energy nodes as $UAV_{MPR}$. This is because MPRs have high communication overhead and require a lot of energy. Thus, this parameter leads to energy saving and higher lifetime. Each UAV loses a certain value of its energy for sending and receiving data or doing internal operations such as computing, linking, updating. Remaining energy is equal to the UAV energy level after doing these operations. If the remaining energy is lower than the threshold ($E_{j}^{Min}$), UAV cannot be selected as $UA{{V}_{MPR}}$ to participate in the routing process. To choose $UA{{V}_{MPR}}$, each node acquires the remaining energy of its neighbors by exchanging the Hello message. Assume that $E_{j}^{Max}\left( t \right)$ is the primary energy of $UA{{V}_{j}}$ at the moment *t*. The remaining energy of $UAV_{j}$ ($E_{j}^{Residual}$) is obtained after a time interval $\tau$ using Eq. (13):
    13 $$\begin{aligned} E_{j}^{Residual}\left( t+\tau \right) =E_{j}^{Max}\left( t \right) -E_{j}^{Consumed}\left( t+\tau \right) , \end{aligned}$$
    where, $E_{j}^{Consumed}\left( t+\tau \right)$ indicates the consumed energy of $UA{{V}_{j}}$ at time interval $\tau$. This parameter is due to sending, receiving data and internal operations done by $UA{{V}_{j}}$ in this interval. Also, the consumed energy for receiving *l* bits, namely $E_{j}^{Received}\left( t+\tau \right)$ and the consumed energy for sending *l* bits, namely $E_{j}^{Send}\left( t+\tau \right)$ (when the Euclidean distance between $UAV_{j}$ and $UAV_{i}$ is equal to ${{d}_{ij}}$) are calculated according to Eqs. (14) and (15), respectively:
    14 $$\begin{aligned} E_{j}^{Received}\left( t+\tau \right) =l\times {E_{elec}}, \end{aligned}$$
    15 $$\begin{aligned} E_{j}^{Send}\left( t+\tau \right) =l\times {E_{elec}}+l\times {{\left( {d_{ij}} \right) }^{2}}\times {E_{amp}}. \end{aligned}$$
    So that $E_{elec}$ is the energy consumed by transceiver circuit for a single bit, $d_{ij}$ is the Euclidean distance between $UAV_{i}$ and $UAV_{j}$. Additionally, $E_{amp}$ indicates the energy required by amplifier. In addition, the energy consumption of UAVs for doing internal operations is displayed as $E_{j}^{Computing}\left( t+\tau \right)$. Therefore, the consumed energy of $UAV_{j}$ in a time interval $\tau$ is equal to the total energy consumption of this node for sending/receiving data and internal operations. It is calculated through Eq. (16):
    16 $$\begin{aligned} \begin{array}{l} E_{j}^{Consumed}\left( t+\tau \right) = \sum \limits _{t\in \tau }{E_{j}^{Send}\left( t+\tau \right) +E_{j}^{Received}\left( t+\tau \right) +E_{j}^{Computing}}\left( t+\tau \right) . \end{array} \end{aligned}$$
    As a result, Eq. (13) is written as follows:
    17 $$\begin{aligned} \begin{array}{l} E_{j}^{Residual}\left( t+\tau \right) =E_{j}^{Max}\left( t \right) - \sum \limits _{t\in \tau }{E_{j}^{Send}\left( t+\tau \right) +E_{j}^{Received}\left( t+\tau \right) +E_{j}^{Computing}}\left( t+\tau \right) \end{array}. \end{aligned}$$
  - ${\textit{CQ}}_{\textit{i,j}}$ This parameter is calculated using Eq. (10). When $CQ_{i,j}$ is high, this means that the communication between $UAV_{i}$ and $UAV_{j}$ is more stable.
  - ${\textit{D}}_{\textit{Neighbor}}$ It is the number of two-hop neighbors of $UAV_{i}$ that are covered by $UAV_{j}$. When $D_{Neighbor}$ of $UAV_{j}$ is high, the communication overhead is reduced. $UAV_{i}$ extracts $D_{Neighbor}$ from its neighborhood table.
  - ${\textit{W}}_{\textit{j}}$ It indicates the willingness of $UAV_{j}$ to carry and send traffic to other UAVs, so that $1\le W_{i}\le 7$. $UAV_{i}$ extracts $W_{j}$ from its neighborhood table. When $W_{j}$ is high, $UAV_{j}$ has more chance to be selected as a MPR node.
  Now, we have:
  18 $$\begin{aligned} \begin{array}{l} F_{j}=\left( \frac{E_{j}^{Residual}-E_{Min}}{{E_{Max}}-E_{Min}}\right) +\left( \frac{{CQ_{i,j}}-{CQ_{Min}}}{{CQ_{Max}}-{CQ_{Min}}}\right) +\left( \frac{{D_{Neighbor}}-{D_{Min}}}{{D_{Max}}-{D_{Min}}}\right) +\left( \frac{W_{j}-W_{Min}}{{W_{Max}}-W_{Min}}\right) , \end{array} \end{aligned}$$
  where, $E_{Max}$ is the maximum energy of UAVs in the network. And, $E_{Min}$ is equal to 0. Also, $CQ_{Max}$ is the maximum connection quality in the neighborhood table of $UAV_{i}$ and $CQ_{Min}$ is the minimum connection quality in this table. Furthermore, $D_{Max}$ represents the maximum neighborhood degree in the neighborhood table of $UAV_{i}$ and $D_{Min-Neighbor}$ is the minimum neighborhood degree in this table. Also, $W_{Max}=7$ and $W_{Min}=1$.
- Update the position of fireflies based on $F_{j}$ and using the equations represented in the firefly algorithm (Eqs. 1–7).
- Remove identifiers of all two-hop neighbors from $List_{2Hop-Neighbor}$ when they are covered by at least one MPR.

### Discovering network topology

**Figure 5:**
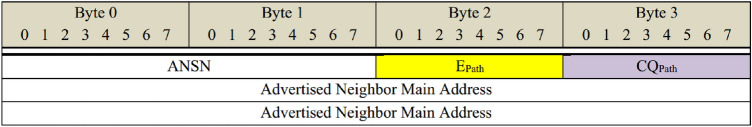
*TC message* format.

In this phase, $UAV_{MPR}$ nodes broadcast the topology control (TC) message in the network. When a $UAV_{MPR}$ receives this message, it rebroadcasts *TC message* in the network. *TC message* format is shown in Fig. 5. We add two fields, including $E_{Path}$ and $CQ_{Path}$, to this message. These fields are shown in Fig. 5 with yellow and purple colors, respectively. In the following, we explain how calculate these two parameters.

- $E_{Path}$ This field indicates the route energy. It means the lowest energy of $UAV_{MPR}$ nodes in one path. In general, the energy of $Route_{i}$ ($E_{Path_{i}}$) is calculated according to Eq. (19):
  19 $$\begin{aligned} E_{Path_{i}}=\underset{UAV_{MPR_{j}}\in Route_{i}}{\mathop {\min }}\,\left( {E_{UAV_{MPR_{j}}}} \right) ,\,\,\,\,\,\,j=1,...,{M_{i}}, \end{aligned}$$
  where, $M_{i}$ indicates the number of $UAV_{MPR}$ nodes in $Route_{i}$. And, $E_{UAV_{MPR_{j}}}$ is the residual energy of $UAV_{MPR_{j}}$.
- $CQ_{Path}$ This field indicates the route quality. It is equal to the lowest quality of the link between $UAV_{MPR}$ nodes in a route. In general, the quality of $Route_{i}$ is obtained using Eq. (20):
  20 $$\begin{aligned} CQ_{Path_{i}}=\underset{UAV_{MPR_{j}}\in Route_{i}}{\mathop {\min }}\,\left( CQ_{{UAV_{MPR_{j-1}}},UAV_{MPR_{j}}}\right) ,\,\,\,\,\,\,j=1,...,{M_{i}}, \end{aligned}$$
  where, $M_{i}$ is the number of $UAV_{MPR}$ nodes in $Route_{i}$. In addition, $CQ_{UAV_{MPR_{j-1}},UAV_{MPR_{j}}}$ indicates the connection quality of the link between $UAV_{MPR_{j}}$ and its previous hop node, namely $UAV_{MPR_{j-1}}$ in $Route_{i}$.

After broadcasting the *TC message*, each node in the network creates a topology table. Note that the topology table template in our scheme is similar to OLSR, but there are two differences. We add two fields, including $E_{Path}$ and $CQ_{Path}$ to the topology table. The topology table format is presented in Table 2. In the following, we explain the fields of this table. Note that the topology table is used to calculate the routing table, which is explained in “Computing different routes” section.

**Table 2:** Topology table.

$UAV_{Destination}$	$UAV_{Last{\text{-}}hop}$	*ANSN*	$E_{Path}$	$CQ_{Path}$	$Time_{Valid}$

- $UAV_{Destination}$ This field indicates the main address of the neighboring node. It is obtained from the *Advertised Neighbor Main Address* field in the *TC message*.
- $UAV_{Last{\text{-}}hop}$ This field indicates the address of $UAV_{MPR}$, which sends the *TC message*.
- *ANSN* This field is the sequence number that is obtained from the *TC message*. It is used to detect duplicated *TC messages*.
- $E_{Path}$ In the topology table, we record the route energy. It is achieved from the *TC message*.
- $CQ_{Path}$ In the topology table, we record route quality, which is obtained from the *TC message*.
- $Time_{Valid}$ This field indicates the validity time of this entry in the topology table.

**Figure 6:**
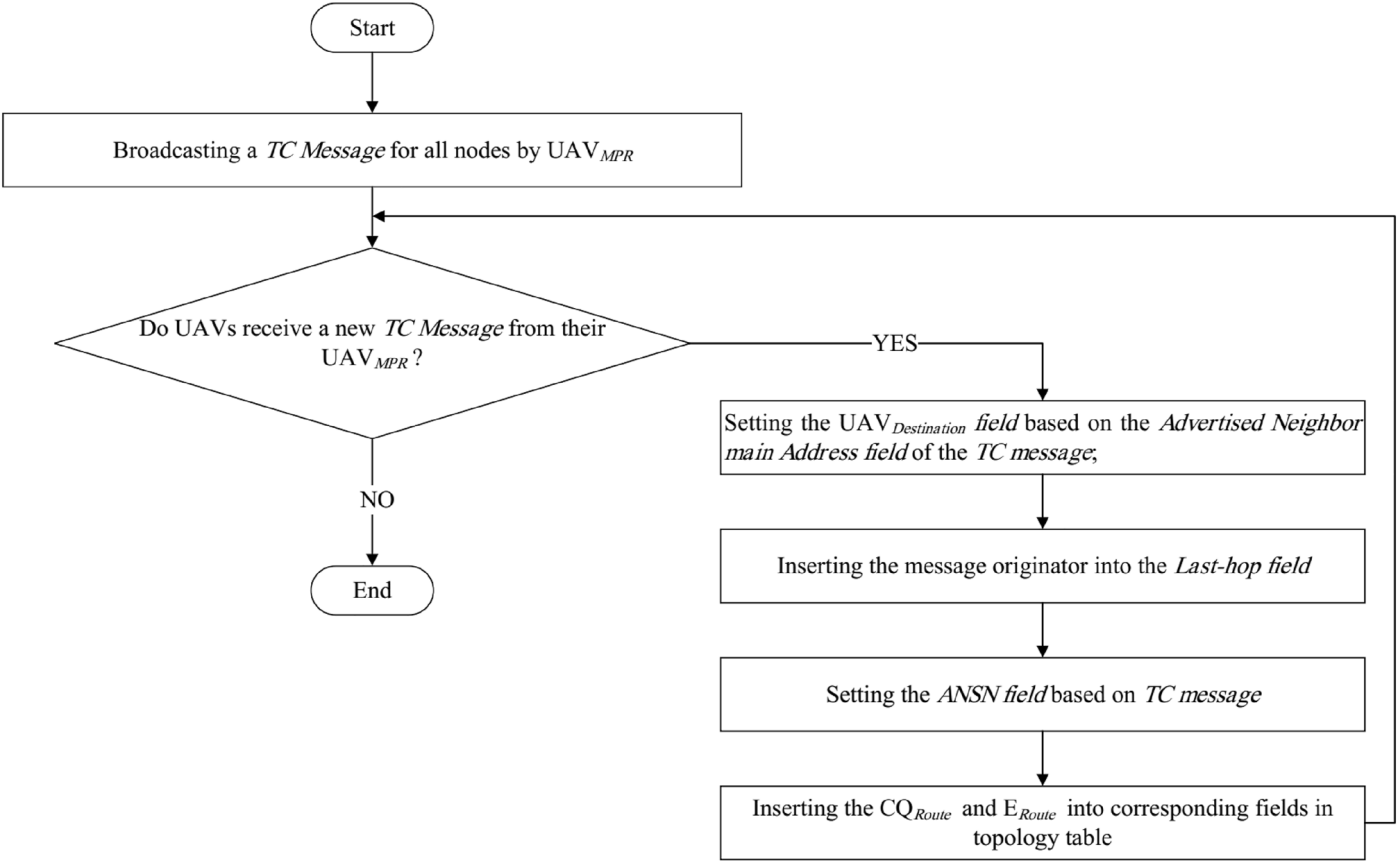
Flowchart of the network topology discovery.

Figure 6 represents the flowchart of the network topology discovery.

### Computing different routes

In the route calculation process, OLSR only considers hop count. However, the proposed method considers several parameters for this process. They include hop count, route energy, and route quality. As a result, our approach creates more stable routes. Table 3 shows the routing table stored in the memory of the nodes. Note that if the neighborhood table or the topology table have changed, the routing table must be re-calculated. Initially, we introduce the fields of the routing table.

**Table 3:** Routing table.

$UAV_{Destination}$	$UAV_{Next{\text{-}}hop}$	$Hop_{Count}$	$Interface\_address$

- $UAV_{Destination}$ This field is the destination node address.
- $UAV_{Next{\text{-}}hop}$ This field indicates the next-hop address to reach the destination node.
- $Hop_{Count}$ This field represents the number of hops from the current node to the destination node.
- $Interface\_address$ This field is the local interface address to send data to the next-hop node.

**Figure 7:**
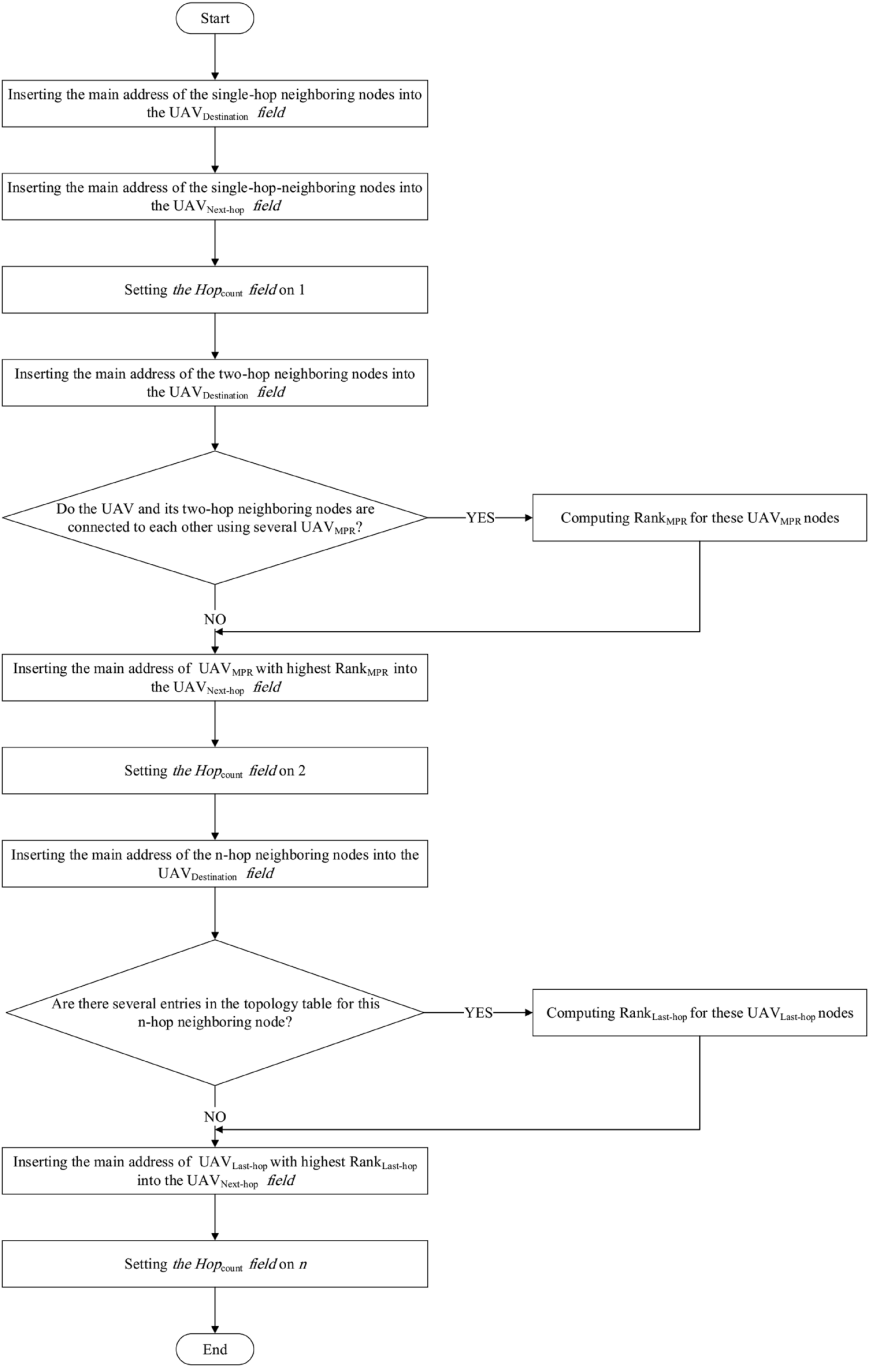
Flowchart of calculating routing table.

In the following, we describe steps for calculating the routing table. Furthermore, it is expressed in Fig. 7.

- $UAV_{i}$, ($i=1,...,N$), extracts the address of its single-hop neighbors from the neighborhood table and inserts them as the destination node into the routing table. In this case, the $UAV_{Next{\text{-}}hop}$ field is also equal to the node address.
- $UAV_{i}$ extracts the address of the two-hop neighbors from the neighborhood table and inserts them as the destination node into the routing table. Note that $UAV_{i}$ records the address of $UAV_{MPR}$ related to the two-hop neighbor in the $UAV_{Next{\text{-}}hop}$ field. If $UAV_{i}$ checks its neighborhood table and finds that $UAV_{2{\text{-}}hop{\text{-}}neighbor}$ is related with several $UAV_{MPR}$ nodes, then $UAV_{i}$ calculates $Rank_{MPR}$ for these $UAV_{MPR}$ nodes and insert the node with the most $Rank_{MPR}$ as the next-hop node into the $UAV_{Next{\text{-}}hop}$ field. This rank is calculated using Eq. (21):
  21 $$\begin{aligned} Rank_{MPR}={w_{1}}\left( \frac{{W_{i}}}{7}\right) +{w_{2}}\left( \frac{{E_{UAV_{MPR}}}}{{E_{Max}}} \right) +{w_{3}}\left( \frac{{{{\bar{CQ}}}_{MPR}}}{{CQ_{Max}}}\right) , \end{aligned}$$
  where, $W_{i}$ is the willingness of a node for transmitting traffic to other nodes in the network and $0\le W_{i}\le 7$. $E_{UAV_{MPR}}$ is the remaining energy of $UAV_{MPR}$. $E_{Max}$ indicates the primary energy of UAVs. Also, $CQ_{Max}$ indicates the maximum connection quality of the neighboring nodes of $UAV_{i}$. Note that all of this information is obtained from the neighborhood table. Also, $\bar{CQ}_{MPR}$ is calculated using Eq. (22):
  22 $$\begin{aligned} {{\bar{CQ}}_{MPR}}=\frac{{CQ_{i,MPR}}+{CQ_{MPR,2{\text {-}}hop{\text {-}}neighbor}}}{2}, \end{aligned}$$
  where, ${CQ_{i,MPR}}$ is the quality of the link between $UAV_{i}$ and $UAV_{MPR}$. ${CQ_{MPR,2{\text {-}}hop{\text {-}}neighbor}}$ indicates the quality of the link is between $UAV_{MPR}$ and $UAV_{2{\text {-}}hop{\text {-}}neighbor}$. Also, $w_{1}$, $w_{2}$, and $w_{3}$ are weight coefficients, so that $\sum \limits _{i=1}^{3}{{w_{i}}}=1$ and $w_{1}=w_{2}=w_{3}=\frac{1}{3}$.
- $UAV_{i}$ inserts the address of multi-hop nodes ($UAV_{n{\text {-}}hop{\text {-}}neighbor}$) as the destination node into its routing table. First, $UAV_{i}$ checks its topology table. If there are several entries in the topology table for $UAV_{n{\text {-}}hop{\text {-}}neighbor}$, then $UAV_{i}$ inserts the $UAV_{Last{\text {-}}hop}$ node with the highest $Rank_{Last{\text {-}}hop}$ as the next-hop node into the $UAV_{Next{\text {-}}hop}$ field. $Rank_{Last-hop}$ is calculated using Eq. (23):
  23 $$\begin{aligned} Rank_{Last-hop}={w_{1}}\left( \frac{{W_{i}}}{7}\right) +{w_{2}}\left( \frac{{E_{Path}}}{{E_{Max}}}\right) +{w_{3}}\left( \frac{{CQ_{Path}}}{{CQ_{MAX{\text {-}}Path}}}\right) , \end{aligned}$$
  where, $W_{i}$ is the willingness of a node for transmitting traffic to other nodes in the network and $0\le W_{i}\le 7$. $E_{Path}$ and $CQ_{Path}$ are the route energy and the route quality obtained from the topology table. Also, $CQ_{MAX{\text {-}}Path}$ is the maximum quality of different routes for $UAV_{n{\text {-}}hop{\text {-}}neighbor}$. It is extracted from the topology table. Also, $w_{1}$, $w_{2}$, and $w_{3}$ are weight coefficients, so that $\sum \limits _{i=1}^{3}{{w_{i}}}=1$ and $w_{1}=w_{2}=w_{3}=\frac{1}{3}$.

## Simulation and evaluation of results

In this section, we simulate our method using NS3 software. To simulate the movement of drones in the network, we use the random waypoint mobility (RWP). In RWP, UAVs are stopped at a fixed time interval called the stop period. When this time is terminated, the node selects a random position and a random speed in the simulation environment. Then, it moves toward this position. After the UAV reaches this position, it waits for the stop period to restart its movement. This process continues during all simulation times. In RWP, the most important advantage is its simple implementation. For this reason, most routing methods use this model to simulate the movement of drones in FANET. In the simulation process, assume that the network size is equal to $500 \times 1500 \times 1500$ m$^{3}$. Also, the number of UAVs in the network is between 10 and 50. These nodes are randomly and uniformly distributed in the network. The simulation time is 200 seconds. Table 4 presents the most important simulation parameters in summary. Then, we compare the simulation results of our proposed routing scheme with the two methods, including G-OLSR^33^ and OLSR^32^. They are evaluated in terms of delay, routing overhead, packet delivery rate, throughput, and energy consumption.

**Table 4:** Simulation parameters.

Parameter	Value
Simulator	NS3
MAC protocol	IEEE 802.11a
Network size (m$^{3}$)	$500 \times 1500 \times 1500$ m$^{3}$
Mobility model	RWP
The number of nodes	10–50
Speed of nodes (m/s)	25
Communication ranges (m)	250
Initial energy of UAVs (J)	2000
Data rate (Mbps)	3
Packet interval (s)	0.2
Simulation time (s)	200

### End-to-end delay

This parameter divides the time required for transferring the message from the source node to the destination node by the total number of nodes in a path. It consists of the transmission delay (${{D}_{trans}}$), the propagation delay of the signal (${{D}_{prop}}$), and the queuing delay (${{D}_{queue}}$) in the entire network. This metric can be calculated using Eq. (24).

24 $$\begin{aligned} {{D}_{end{\text {-}}to{\text {-}}end}}=N\left( {{D}_{trans}}+{{D}_{prop}}+{{D}_{proc}}+{{D}_{queue}} \right) . \end{aligned}$$

**Figure 8:**
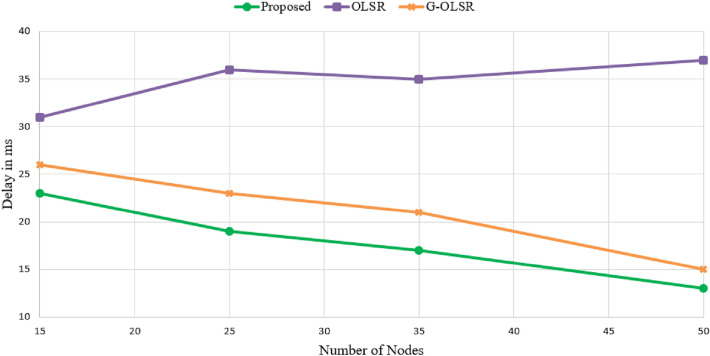
Comparison of different routing methods in terms of end-to-end delay.

Figure 8 compares the end-to-end delay in different routing methods. As shown in this figure, our method has the lowest delay in comparison with other routing schemes. In Fig. 8, our scheme reduces the end-to-end delay by 15.29% and 48.20% compared to G-OLSR and OLSR, respectively. The proposed method considers two parameters, including route quality and route energy to make more stable paths. This reduces the number of route failures. Furthermore, it decreases the updating time of the routing table, this helps our scheme to reduce the transmission delay. In contrast, OLSR considers only the number of hops for creating different paths. It is not enough. Also, G-OLSR does not consider energy and link quality in the route establishment process. In G-OLSR, paths are determined by a greedy scheme, which is based on the distance from nodes to the destination. If the greedy method cannot find a proper path to the destination, G-OLSR switches to the route recovery process and selects a node with the minimum angle toward the destination as the next-hop node. This routing strategy reduces the end-to-end delay properly. However, it has weaker performance than our scheme. On the other hand, we design a new approach based on the firefly algorithm for selecting MPRs. According to the MPR-FA algorithm, when nodes have high energy and more stable links and more neighborhood degree, they obtain more fitness to be selected as MPRs. As a result, the MPR-FA scheme helps our approach to select more stable nodes as the MPR node. This increases the network stability. As a result, the paths formed between nodes are valid for more time. This successfully reduces delay in our scheme compared to other methods. According to Fig. 8, when the number of nodes in the network is high, the delay is reduced in our scheme and G-OLSR. While this issue does not affect OLSR and even increases the delay. This is because our method and G-OLSR select optimal routes to the destination. These methods are more consistent with FANETs, and they can manage rapid changes in the topology and high-speed nodes, which fail communication links. While OLSR is not such and cannot manage this condition. As a result, it has a weak performance in FANET.

### Routing overhead

**Figure 9:**
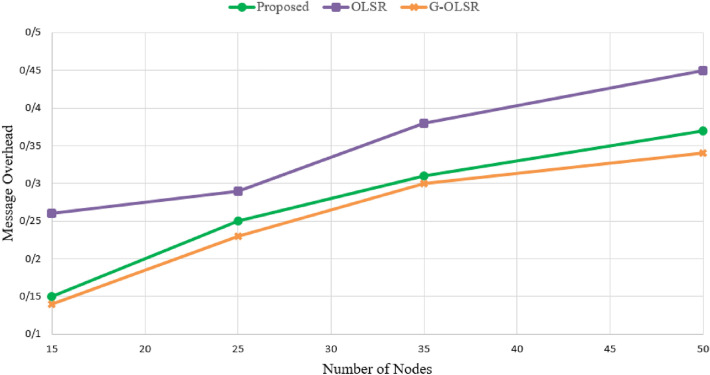
Comparison of different routing methods in terms of routing overhead.

This parameter indicates the ratio of Beacon messages to the total number of messages in the network. Figure 9 shows routing overhead in different schemes. As shown in this figure, our method reduces routing overhead compared to OLSR, but it has a weaker performance than G-OLSR. In Fig. 9 our routing method improves the routing overhead in comparison with OLSR (21.74%), while it has a higher routing overhead compared to G-OLSR (6.93%). This is because the proposed scheme uses a firefly algorithm-based mechanism for selecting MPRs. This mechanism increases computational and communication overheads. On the other hand, G-OLSR considers a greedy mechanism for selecting the next-hop node. A combination of the greedy scheme and OLSR helps G-OLSR to reduce routing overhead. Another important point is that in all methods, when increasing the number of nodes in the network, the routing overhead is also increased because in this case, the schemes exchange more *Hello messages* and *TC messages* to update neighborhood table and topology table in nodes.

### Packet delivery rate

**Figure 10:**
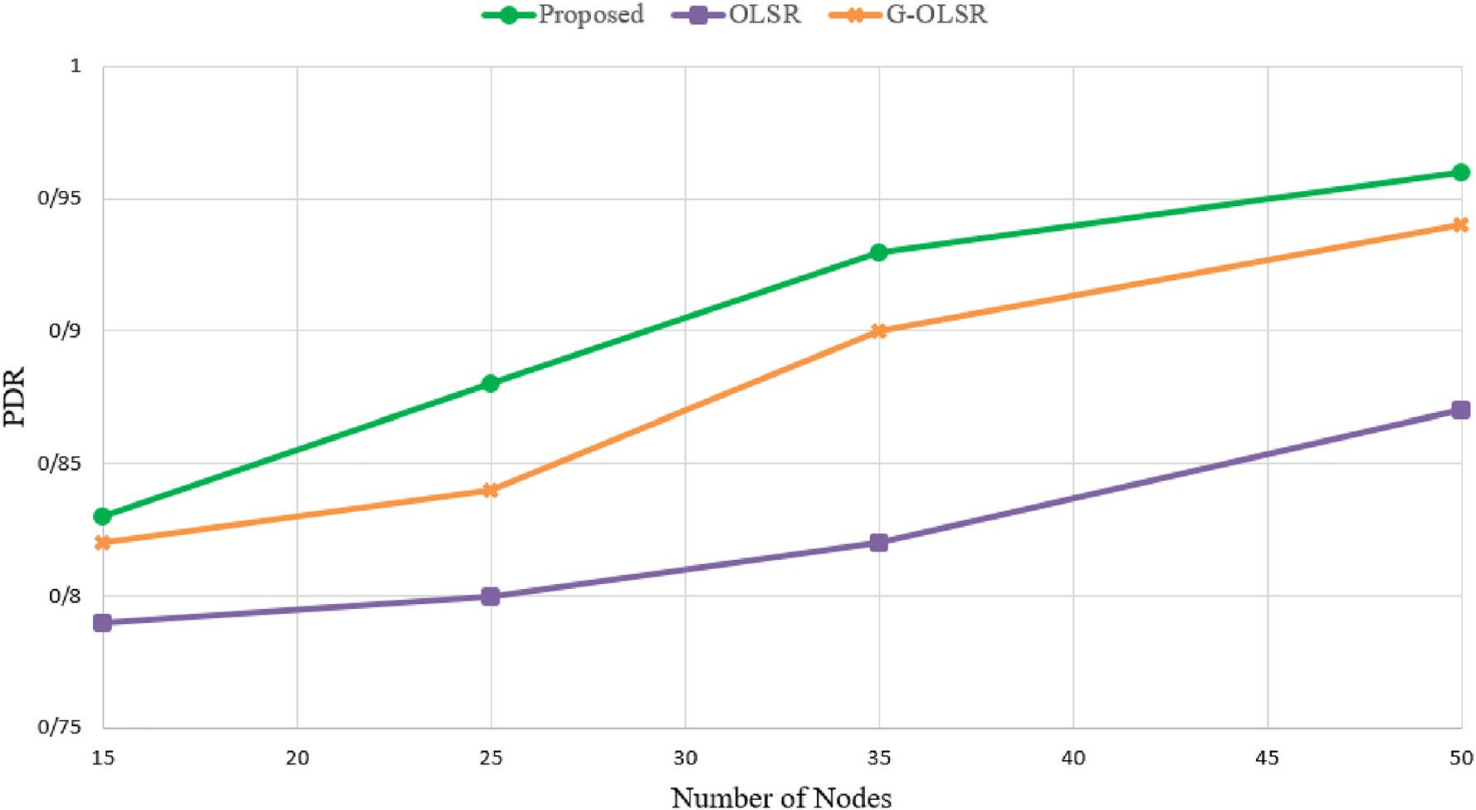
Comparison of different routing methods in terms of PDR.

This parameter indicates the ratio of messages received at the destination to the total messages transmitted in the network. It is calculated based on Eq. (25):

25 $$\begin{aligned} PDR=\frac{\sum \nolimits _{i=1}^{n}{{{P}_{r}}}}{\sum \nolimits _{i=1}^{n}{{{P}_{s}}}}\times 100, \end{aligned}$$

where, ${{P}_{r}}$ is packets received by the destination UAV and ${{P}_{s}}$ is packets sent by the source UAV. As shown in Fig. 10, our method has the best PDR compared to G-OLSR and OLSR. In Fig. 10, the proposed routing scheme increases PDR by 2.86% and 9.76% in comparison with G-OLSR and OLSR methods, respectively. When PDR has a better value, this means that routes are more stable. In the proposed method, we focus on the route stability. We consider the energy of nodes in the route calculation process and the MPR selection process so that high-energy nodes have more chance to participate in the route formation process. Also, we focus on the link quality and estimate the connection quality based on the ratio of sent/received of hello packets and connection time to select routes with high quality link for sending data packets. In contrast, OLSR does not consider these parameters. G-OLSR uses a greedy routing process only based on distance. Also, it focuses on only one parameter, namely motion angle in the recovery phase. For these reasons, our method has better performance than OLSR and G-OLSR in terms of PDR. According to Fig. 10, another point is that PDR in different schemes increases when increasing the number of nodes in the network, because it reduces the probability of route failures.

### Throughput

**Figure 11:**
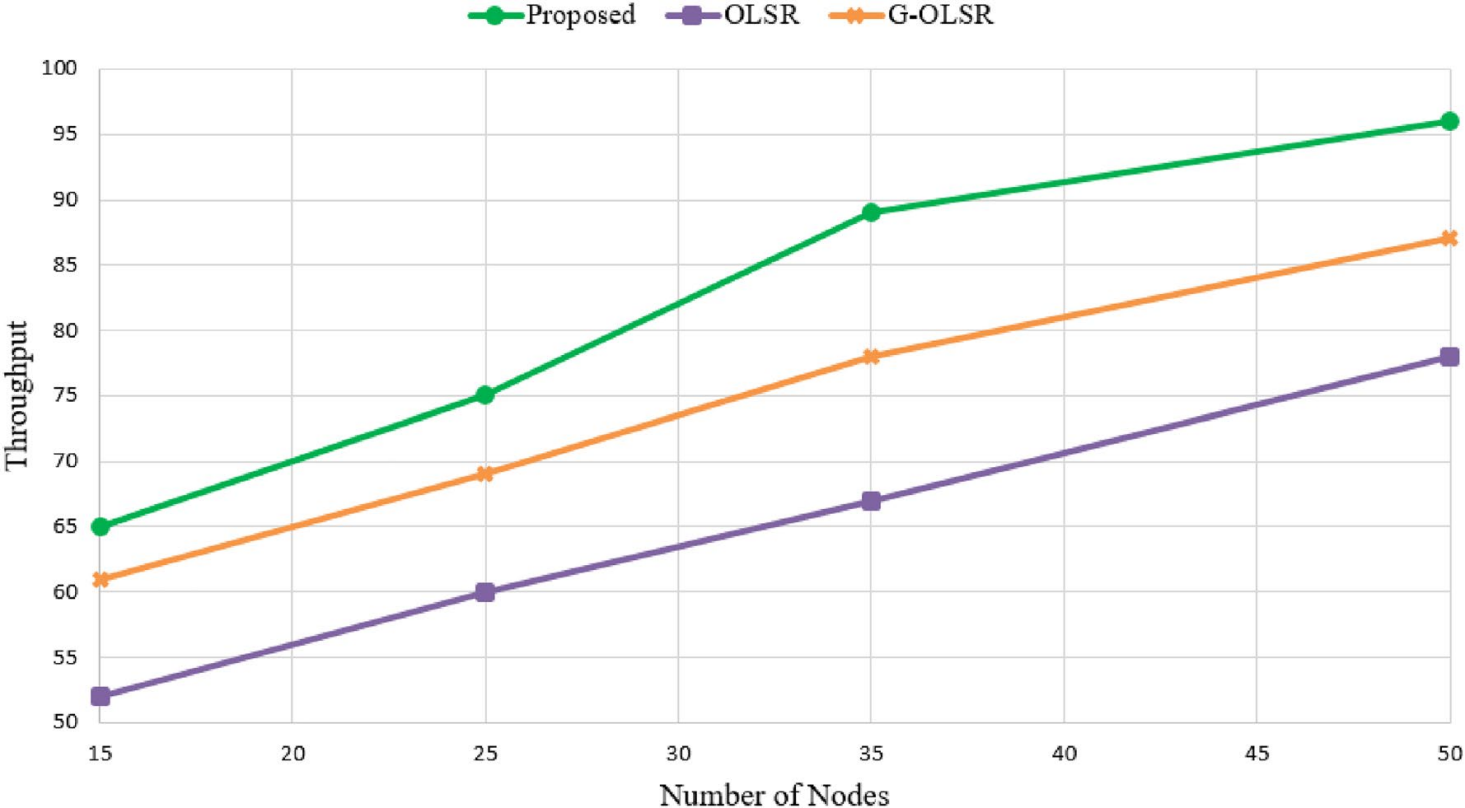
Comparison of different routing methods in terms of throughput.

This parameter is the ratio of messages delivered to the destination to the total time required to reach the destination. As shown in Fig. 11, our proposed approach improves throughput by 10.17% and 26.46% compared to G-OLSR and OLSR, respectively. This proves that our method form suitable paths between nodes in the network. This is because low-energy nodes do not participate in the route creation process and have a very small chance to be selected as MPR nodes. As a result, routes have more energy and higher lifetime. As a result, our scheme prevents unstable paths. These reasons improve throughput in our method compared to other methods. As shown in Fig. 11, when increasing nodes in the network, throughput is also increased in all methods because this increases the chance to find the suitable paths between nodes and improves connections between nodes in the network.

### Energy consumption

**Figure 12:**
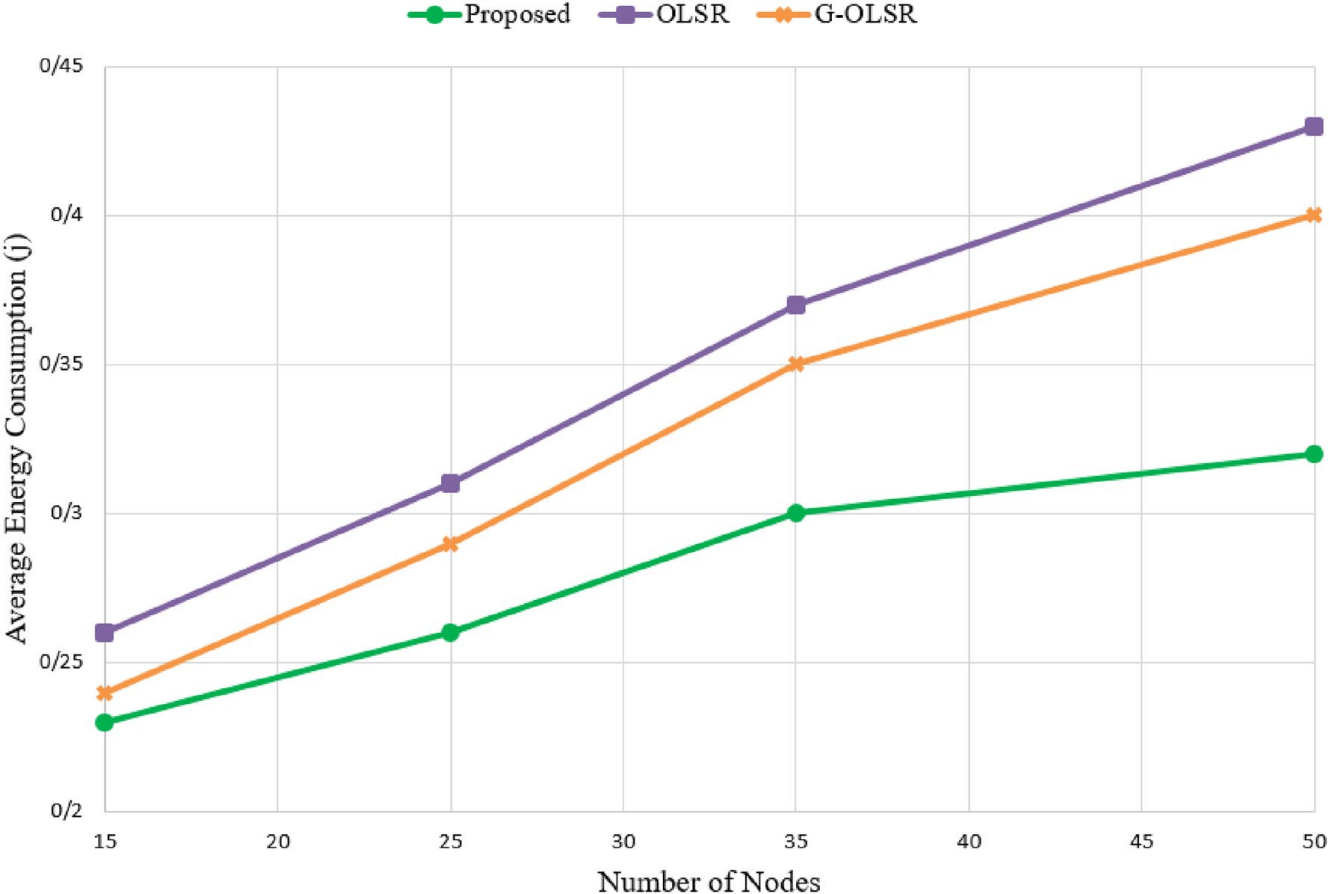
Comparison of different routing methods in terms of energy consumption.

In Fig. 12, the average energy consumption is compared in different methods. As shown in this figure, our method has the lowest energy consumption than other schemes. Based on Fig. 12, the proposed method reduces energy consumption by 13.28% and 18.98% compared with G-OLSR and OLSR, respectively. This is because we consider the energy parameter for calculating paths and selecting MPR nodes, while OLSR and G-OLSR do not pay attention to this parameter in their routing process. Also, our scheme forms more stable paths because this method considers energy consumption and connection quality. Therefore, paths are valid for more time. As a result, our method has a lower need for updating routing tables. This helps our scheme to improve energy consumption. Another point is that in all methods, energy consumption is boosted when increasing the number of nodes in the network. This is because in this case, these routing methods need to exchange more control messages (Hello and TC messages) in the network. This increases their communication overhead and energy consumption.

## Conclusion

In this paper, we proposed an energy-aware routing scheme using firefly algorithm for FANETs. In our scheme, a new approach is introduced for estimating the connection quality. It is estimated based on the ratio of sent/received of hello packets and connection time. Then, we presented the MPR-FA algorithm to choose the best set of MPRs in the network. In this algorithm, a fitness function is defined based on four parameters, including energy, connection quality, neighborhood degree, and willingness. Finally, our scheme calculates the paths between nodes based on path energy and path quality. NS3 software is used for simulating our scheme and evaluating its performance. The RWP mobility model is considered for simulating the movement of UAVs in the simulation process. We compare our scheme with G-OLSR and OLSR. The simulation results show that our method has a successful performance compared to G-OLSR and OLSR in terms of end-to-end delay, PDR, throughput, and energy consumption. However, our approach has more routing overhead compared to G-OLSR. In the future research direction, we must evaluate our scheme with other mobility models and more scenarios to highlight the efficiency of our method. Also, we try to present a multi-path routing scheme using machine learning (ML) techniques in FANET for improving network fault tolerance.

## Data Availability

All data generated or analyzed during this study are included in this published article.
